# Using the Atherogenic Index of Plasma to Estimate the Prevalence of Ischemic Stroke within a General Population in a Rural Area of China

**DOI:** 10.1155/2020/7197054

**Published:** 2020-12-14

**Authors:** Chang Wang, Zhi Du, Ning Ye, Songyue Liu, Danxi Geng, Pengbo Wang, Yingxian Sun

**Affiliations:** Department of Cardiovascular Medicine, The First Hospital of China Medical University, Shenyang, Liaoning 110001, China

## Abstract

**Objective:**

To investigate the relationship between the atherogenic index of plasma (AIP) and ischemic stroke.

**Design:**

We collected a range of data from 11,495 residents (aged ≥35 years; 54.28% female) residing in rural areas of northeast China between January and August 2013, including fasting lipid profile and anthropometric parameters. Logistic regression models were used to evaluate the correlation between AIP and ischemic stroke. Category-free analysis was used to determine whether AIP enhanced our capacity to estimate ischemic stroke.

**Results:**

Irrespective of gender, AIP was independently associated with the occurrence of ischemic stroke. The prevalence of ischemic stroke increased significantly from the lowest quartile to the highest quartile (females: 10.5%-48.7%, *P* < 0.001; males: 22.0%-36.5%, *P* = 0.08). After adjusting for age, gender, income, education, smoking, drinking, exercise, hypertension, body mass index (BMI), diabetes, atrial fibrillation, and a family history of stroke, we found that for every 1 standard deviation (SD) increase in AIP, there was a 34.8% and 20.9% increase in the prevalence of stroke for females and males, respectively. Curve fitting was also used to evaluate the linear relationship between AIP and the occurrence of ischemic stroke. Category-free analysis indicated that AIP significantly enhanced our ability to estimate ischemic stroke in both females (NRI (95% confidence interval (CI)): 0.188 (0.105-0.270)) and males (NRI (95% CI): 0.175 (0.017-0.333)).

**Conclusion:**

Analyses detected a significant and positive linear relationship between AIP and the prevalence of ischemic stroke. This relationship was independent of a range of conventional risk factors.

## 1. Introduction

Stroke is a primary risk factor for disability and the second cause of death in subjects aged >60 years [1]. The incidence of stroke in the population of China has increased gradually over the last three decades and is estimated to affect 2.4 million subjects each year [2]. The prevalence of ischemic stroke is particularly high in low-income groups in rural areas of China [3]. Consequently, it is very important to screen high-risk groups of subjects in rural areas of China and take appropriate preventative measures in order to control the occurrence of stroke events. Previous studies have also shown that small, dense, low-density lipoprotein (sdLDL) may contribute to the formation of carotid plaques; these structures exhibit a significant relationship with intima-media thickness in the carotid artery [4]. Patients with high levels of sdLDL are more likely to develop atherosclerosis, thereby increasing the risk of coronary artery disease, myocardial infarction, carotid artery disease, and ischemic stroke. Determining the plasma levels of sdLDL is therefore an efficient method with which to predict the risk of ischemic stroke. However, existing methods for measuring sdLDL are associated with a range of limitations, including high running costs. Consequently, such methods cannot be applied in low-income rural areas. Therefore, there is an urgent need to identify cost-effective and reproducible markers as alternatives to sdLDL so that risk stratification for ischemic stroke can be effectively improved.

Several potential markers have emerged over recent years. For example, the atherogenic index of plasma (AIP) is a useful indicator for plasma atherosclerosis and is easily calculated as the logarithm of the molar ratio of triglyceride to high-density lipoprotein cholesterol (TG to HDL-C) [5]. Previous studies have evaluated the relationship between AIP, a suitable alternative to sdLDL [6], and certain clinical diseases including coronary heart disease [7], hypertension, diabetes, metabolic syndrome, and hyperuricemia [7–9]. However, it remains unclear as to whether AIP can optimize risk stratification for ischemic stroke in a manner that is independent of other conventional risk factors. Moreover, the findings of previous studies have yet to be validated in large general populations. Therefore, the purpose of the present study was to investigate whether higher levels of AIP are associated with ischemic stroke among a general population in rural China.

## 2. Methods

### 2.1. Study Population

Data were acquired from a large cross-sectional population study referred to as the Northeast China Rural Cardiovascular Health Study (NCRCHS) between January and August 2013. This study used a multistage, stratified, random, and clustered, sampling method. The design and principles of the NCRCHS have been described in detail elsewhere [10–12]. A representative group of adults (aged ≥35 years) were recruited from Liaoning Province as research subjects. A total of 14,016 subjects were invited to participate in this study; of these, 11,956 subjects completed the baseline survey (a response rate of 85.3%). Of the 11,956 subjects who completed the baseline survey, 491 lacked data relating to biomarkers and clinical covariates; these subjects were therefore excluded from our analysis. Therefore, 11,495 subjects were incorporated in the final analysis. This research was approved by the Ethics Committee of China Medical University. Following preliminary screening, all subjects were required to provide informed and written consent in order to participate in the study. Informed consent could be acquired from proxies if participants were unable to provide consent themselves; for example, in the case of disability. All data acquisition, storage, and analyses, were carried out in accordance with the approved ethical protocol.

### 2.2. Data Collection and Testing

The process used for data collection was comprehensively described in our previous publication [11, 13, 14]. In brief, cardiologists and trained nurses conducted a questionnaire survey that focused upon health-related behaviors, demographics, and whether the subjects had taken any drugs over the previous two weeks. We also collected data relating to lifestyle risk factors, education, family income, and medical history. Blood pressure measurements were acquired from two trained nurses after a 5-minute rest period using an automatic electronic sphygmomanometer (HEM-907; Omron, Kyoto, Japan). Blood pressure readings were recorded three times, at 1- to 2-minute intervals; the three readings were then averaged. Then, subjects were asked to remove their shoes and any heavy objects so that we could determine a range of anthropometric indices. We use a calibrated stadiometer to measure standard height to the nearest 0.1 cm. We also determined standard weight to the nearest 0.1 kg using calibrated digital scales. Samples of blood were taken from the cubital veins of each subject (after at least 12 hours of fasting) to determine the plasma levels of fasting glucose (FPG), TG, and HDL-C. A complete description of the procedures used for storing blood samples, and the measurement of laboratory indicators, has been published elsewhere [12, 13].

### 2.3. Definitions

Body mass index (BMI) was defined as body weight divided by the square of height. AIP was defined as the logarithm of the molar ratio of TG to HDL-C [6]. Hypertension was defined as a blood pressure level of at least 140/90 mmHg, for any subject using antihypertensive drugs. Cerebrovascular diseases, such as ischemic stroke and hemorrhagic stroke, were diagnosed by neurologists following the examination of computed tomography (CT) and magnetic resonance imaging (MRI) data in accordance with World Health Organization (WHO) criteria [11]. Subjects were referred to as being diabetic if their fasting blood glucose (FPG) level was ≥7.0 mmol/L, if they had a self-reported history of a previous diagnosis of diabetes, or if they were currently receiving hypoglycemic therapy [12].

### 2.4. Data Analysis

Our analysis involved both genders. Continuous variables are expressed as mean ± standard deviation (SD), while categorical variables are described as frequencies (percentages). The Student *t*-test or chi-squared test was used to compare the ischemic stroke group with the nonischemic stroke group. AIP was analyzed as a continuous variable (increasing per SD) and was then classified into quartiles; thus, the AIP population was divided into four groups. Following the adjustment of data for confounding variables, we used multivariate logistic regression to determine the independent association between AIP and ischemic stroke. A number of mixed variables were adjusted, including age, gender, race, education, income, smoking and drinking, exercise, BMI, hypertension, diabetes, a family history of stroke, and atrial fibrillation. Results are expressed as an odds ratio (OR) with 95% confidence intervals (95% CIs). Finally, restricted cubic splines were used to investigate the relationship between AIP and the risk of ischemic stroke. We also calculated reclassification improvement (NRI) data (conventional risk factors only *vs.* conventional risk factors + AIP) to evaluate the extent to which AIP improved our ability to predict risk in comparison with more traditional risk factors. All statistical analyses were performed by SPSS 25.0 software (IBM Corporation), the R statistical software package (http://www.r-project.org, R Foundation), and EmpowerStats software (http://www.empowerstats.com, X&Y Solutions, Inc. (Boston, Massachusetts). A two-tailed *P* value < 0.05 was considered to be statistically significant.

## 3. Results

A total of 11,495 people participated in this study (54.3% were female). The mean ages of the female and male participants were 53.2 ± 10.3 years and 63.13 ± 8.31 years, respectively. As shown in Table 1, the prevalence of ischemic stroke in males and females was 3.0% and 3.1%, respectively. Table 1 shows a comparison of the clinical and biochemical risk factor profiles between the two genders. For both genders, patients with ischemic stroke were older than nonischemic stroke patients. In addition, patients with ischemic stroke tended to have lower educational levels and lower family incomes than nonischemic stroke patients. Some patients with ischemic stroke exhibited higher levels of TG, TC, LDL, and GLU. We also found that BMI, SBP, and DBP were significantly higher in patients with ischemic stroke than in patients without ischemic stroke (*P* < 0.05).

**Figure 1:**
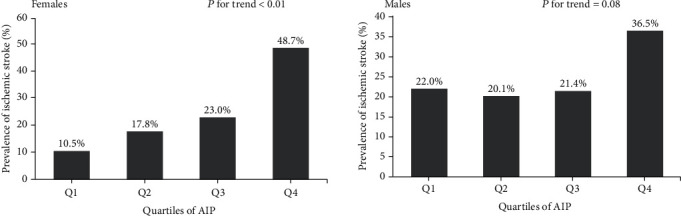
The prevalence of ischemic stroke, as shown by atherogenic index of plasma (AIP) quartiles. In females, the risk of ischemic stroke increased proportionally with ascending AIP quartiles (*P* < 0.05).

Figure 1 shows a comparison between the four AIP quartiles for ischemic stroke rates between the two genders. The prevalence of ischemic strokes showed a continuously increasing trend with increasing AIP quartile. When comparing the top and bottom quartiles, there was a 4.700-fold change in the prevalence of ischemic stroke in female patients; this trend was both linear and significant (*P* < 0.05). Similarly, there was a 1.723-fold change for male patients. These results indicated that AIP was positively correlated with the prevalence of ischemic stroke.

**Figure 2:**
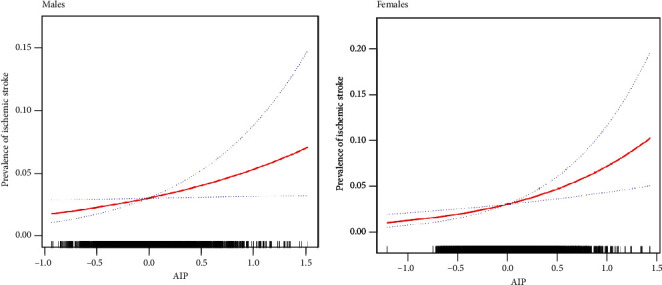
Restricted cubic splines were used to explore the association between AIP and ischemic stroke in males and females after adjusting for age, race, education level, family income, physical exercise, current smoking and drinking status, BMI, hypertension, DM, family history of stroke, and atrial fibrillation. The solid line indicates the estimated risk of ischemic stroke while the dotted lines serve as 95% confidence intervals. For both genders, there was a linear association across the entire range of AIP.

To investigate the relationship between AIP and the prevalence of ischemic stroke between male and female patients, we performed smooth curve fitting, as shown in Figure 2. This analysis showed that there was a linear relationship between AIP and ischemic stroke for both genders.

Next, we carried out multivariate logistic regression analysis to further define the specific relationship between AIP and the prevalence of ischemic stroke (Table 2). In the original model, for each additional SD unit of AIP, the risk of ischemic stroke in females increased by a factor of 1.740 (CI: 1.515-1.999); the risk of ischemic stroke in males increased by a factor of 1.253 (CI: 1.087-1.443). This association was still evident even after adjustment for age, gender, income, education, smoking, drinking, exercising, hypertension, BMI, diabetes, atrial fibrillation, and a family history of stroke; the risk of ischemic stroke for females and males with every additional SD unit of AIP was 1.348-fold and 1.209-fold, respectively (*P* < 0.001). This was also verified by the linear trends identified in restricted cubic spline analysis.

Next, we conducted stratified analysis by subgroups that were defined by covariates to confirm that the findings arising from the logistical model were robust with regard to potential confounding factors. We used a range of covariates that are all known risk factors for stroke, including age, HTN, DM, and BMI. All of these analyses were adjusted for age, gender, race, education status, family income, current smoking and drinking status, physical activity, BMI, hypertension, diabetes, family history of stroke, and atrial fibrillation.  Table 3 shows that the risk for ischemic stroke increased as AIP increased and that this relationship was evident irrespective of the subgroupings.

Category-free analysis further indicated that AIP significantly enhanced our ability to predict stroke in both females (NRI (95% CI): 0.255 (0.111-0.400)) and males (NRI (95% CI): 0.175 (0.017-0.333)) (Table 4).

## 4. Discussion

The study focused on a rural population in China and demonstrated that a significantly positive association existed between AIP and ischemic stroke. Furthermore, we found that this relationship was linear and independent of conventional risk factors. Our results also indicated that AIP can significantly improve risk stratification for ischemic stroke, particularly in a low-income rural population. We used univariate analysis and multivariate logistic regression analysis to analyze our cohort of 11,495 participants. Our results showed that AIP was independently associated with the occurrence of ischemic stroke in both genders. Interestingly, we noted differences in the correlation between AIP and ischemic stroke when compared between males and females; this will be discussed later in this article.

AIP is as an inexpensive and easy-to-evaluate marker that can be used to evaluate the progression of atherosclerosis; consequently, AIP represents a useful alternative to sdLDL, which is difficult to detect in clinical practice. In order to fully evaluate this model, we investigated whether the addition of other markers could enhance the existing predictive model. For this part of our analysis, we used category-free analysis. When AIP was used as a supplementary indicator alongside routine risk factors, we found that there was a significant enhancement in our ability to predict ischemic stroke. These results showed that AIP could be used as an efficient tool during early monitoring to predict the risk of ischemic stroke in the general population. We would then be able to intervene in a targeted manner in order to reduce the future possibility of ischemic stroke. In short, our findings provide a significant improvement in our clinical ability to predict ischemic stroke.

Dobiásová and Frohlich were the first to report that AIP was closely related to HDL levels and the size of lipoprotein particles and therefore recommended that AIP could be used as a marker for atherosclerosis [6]. Since this initial proposal, a series of studies have shown that AIP is also associated with coronary heart disease [8], hypertension [15], diabetes [16, 17], metabolic syndrome, and hyperuricemia [18]. For example, the findings of a large observational study demonstrated that AIP was an emerging indicator and was independently correlated with disease severity in the coronary arteries [19]. This previous study suggested that the combination of HDL and AIP was more conducive to predicting the risk of cardiovascular disease. It is also evident that gender should be considered when considering such diseases. A previous cross-sectional study showed that cardiovascular events occurred more frequently in nonobese sedentary Nigerian men than women [20]. Furthermore, some authors have reported that there is a close relationship between AIP and the plasma levels of uric acid [21, 22], and that this relationship is more pronounced in females than males. One previous study showed that postmenopausal women in Bangladesh exhibited high AIP values and that this was significantly associated with cardiovascular disease (CVD) risk factors [23]. However, there appears to be some debate in the existing literature with regard to the specific relationship between AIP and cardiovascular events. For example, one previous study reported that AIP was not an independent risk factor for CVD after adjustment for confounding factors [24]. However, this particular study featured a low sample size and may not therefore be fully representative of the wider scenario. Another study investigated AIP in a Chinese population and reported that AIP is a strong and independent predictor for coronary artery disease in Chinese Han subjects [25]. To our knowledge, our study is the first to report that AIP is independently associated with the risk of ischemic stroke in a general population. Compared with previous studies, our subjects were recruited from a general population in rural areas of China; we also had a large sample size. Previous studies have shown that AIP predicts outcome events that differ between men and women; therefore, we analyzed males and females separately. Interestingly, we found that this relationship was more pronounced in females than males.

The gender differences we observed in our data could be attributed to a wide range of factors, including culture, race, diet, lifestyle, demographic characteristics, and possibly, the criteria used to select subjects. First, it has been well established that there is a large gender-based difference associated with smoking and drinking; men tend to adopt poor lifestyle habits to a greater degree than women and are therefore more prone to ischemic stroke. Second, it is well known that AIP can be influenced by the lack of estrogen and metabolic changes in menopausal women. The equation used to calculate AIP involves TG and HDL-C. Thus, any biochemical index that influences the values of TG and HDL-C can also affect the value of AIP. Previous research has reported marked reductions in TC, LDL-C, TG, and HDL-C, in postmenopausal women in both Nigeria and Bangladesh. Studies carried out in Nigeria showed that AIP values in the female population increased by 0.32 after menopause [26]. Third, different research groups may generate AIP values in an inconsistent manner. Some studies have confirmed that, compared with other human body measurement indicators, BMI is the strongest predictor of AIP [20]. In our study, the overall BMI in women was higher than that in men. These could explain the fact that the correlation between AIP and ischemic stroke in male patients was not as strong as that in female patients.

Our study has some limitations that need to be considered. First, this study featured a cross-sectional design that could only determine the association between AIP and ischemic stroke. Our analysis did not consider a timeline; longitudinal studies are now needed to study the prognostic role of AIP in newly founded ischemic stroke. Second, our study population was recruited from rural China and may therefore differ from developed countries or high-income regions. However, these limitations do not affect the implications of this study with regard to the future generation of strategies to prevent ischemic stroke.

## 5. Conclusion

Our analyses showed that AIP is linearly related to ischemic stroke and could optimize the risk stratification of ischemic stroke among low-income populations in rural areas of northeastern China.

## Figures and Tables

**Table 1:** Characteristics of subjects with ischemic stroke, as stratified by gender.

Variables	Females (*n* = 6240)			Males (*n* = 5225)		
	Nonischemic stroke (*n* = 6049)	Ischemic stroke (*n* = 191)	*P* value	Nonischemic stroke (*n* = 5066)	Ischemic stroke (*n* = 159)	*P* value
Age (y)	53.2 ± 10.30	63.13 ± 8.31	*<0.001*	54.14 ± 10.74	63.71 ± 9.32	*<0.001*
Ethnicity (Han)%	5739 (94.9)	178 (93.2)	*0.302*	4796 (94.7)	151 (95.0)	*0.869*
SBP (mmHg)	139.53 ± 23.61	161.57 ± 27.64	*<0.001*	143.16 ± 22.33	159.30 ± 26.79	*<0.001*
DBP (mmHg)	80.44 ± 11.47	85.78 ± 12.15	*<0.001*	83.63 ± 11.81	87.72 ± 11.90	*<0.001*
Education status			*<0.001*			*<0.001*
Primary	3411 (56.4)	145 (75.9)		2092 (41.3)	97 (61.0)	
Middle	2162 (35.7)	38 (19.9)		2402 (47.4)	50 (31.4)	
High	476 (7.9)	8 (4.2)		572 (11.3)	12 (7.5)	
Family income (CNY/year, %)			*<0.001*			*<0.001*
≤5000	676 (11.2)	49 (25.7)		665 (13.1)	51 (32.1)	
5000-20000	3345 (55.3)	104 (54.5)		2742 (54.1)	80 (50.3)	
>20000	2028 (33.5)	38 (19.9)		1659 (32.7)	28 (17.6)	
Physical activity (%)			*<0.001*			*<0.001*
Low	2546 (42.1)	125 (65.4)		1406 (27.8)	114 (71.7)	
Middle	1179 (19.5)	28 (14.7)		977 (19.3)	19 (11.9)	
High	2324 (38.4)	38 (19.9)		2383 (53.0)	26 (16.4)	
Smoking, *N* (%)	995 (16.4)	32 (16.8)	*0.911*	2909 (57.4)	79 (49.7)	*0.052*
Drinking, *N* (%)	181 (3)	1 (0.5)	*0.015*	2312 (45.6)	40 (25.2)	*<0.001*
Hypertension, *N* (%)	2824 (46.7)	169 (88.5)	*<0.001*	2634 (52.0)	133 (83.6)	*<0.001*
Diabetes, *N* (%)	652 (10.8)	50 (26.2)	*<0.001*	484 (9.6)	31 (19.5)	*<0.001*
AF, *N* (%)	64 (1.1)	10 (5.2)	*<0.001*	52 (1.0)	12 (7.5)	*<0.001*
BMI (kg/m^2^)	24.82 ± 3.77	25.96 ± 3.71	*<0.001*	24.71 ± 3.55	24.97 ± 3.44	*<0.001*
TG (mmol/L)	1.60 ± 1.32	2.24 ± 1.50	*<0.001*	1.65 ± 1.66	1.91 ± 2.23	*<0.001*
TC (mmol/L)	5.28 ± 1.12	5.62 ± 0.97	*<0.001*	5.16 ± 1.05	5.19 ± 1.02	*<0.001*
LDL (mmol/L)	2.96 ± 0.84	3.29 ± 0.84	*<0.001*	2.86 ± 0.79	3.03 ± 0.83	*<0.001*
HDL (mmol/L)	1.41 ± 0.34	1.33 ± 0.30	*<0.001*	1.41 ± 0.43	1.33 ± 0.41	*<0.001*
GLU (mmol/L)	5.84 ± 1.58	6.47 ± 2.07	*<0.001*	5.93 ± 1.62	6.49 ± 2.29	*<0.001*
AIP	−0.133 ± 0.30	0.16 ± 0.31	*<0.001*	−0.14 ± 0.33	0.07 ± 0.35	*<0.001*

Abbreviations: AIP—atherogenic index of plasma; SBP—systolic blood pressure; DBP—diastolic blood pressure; China Yuan: 1 CNY = 0.158 USD; FBG—fasting blood glucose; TG—triglycerides; TC—total cholesterol; LDL-C—low-density lipoprotein cholesterol; HDL-C—high-density lipoprotein cholesterol; CNY—China Yuan.

**Table 2:** Multivariate logistic regression analyses for AIP and ischemic stroke.

Variables	*N*	Odd ratios (95% CI)			
		Crude	*P* value	Model	*P* value
Females			*<0.001*		
AIP (per SD change)	6240	1.740 (1.515-1.999)	*0.002*	1.348 (1.142-1.591)	*<0.001*
Quartiles of AIP					
Q1	1503	1.000 (reference)			
Q2	1579	1.632 (0.935-2.848)	*0.085*	1.202 (0.672-2.150)	*0.534*
Q3	1626	2.062 (1.210-3.515)	*0.008*	1.180 (0.670-2.079)	*0.567*
Q4	1532	4.792 (2.940-7.811)	*<0.001*	2.129 (1.245-3.640)	*0.006*
*P* for trend			*<0.001*		*0.002*
Males					
AIP (per SD change)	5225	1.253 (1.087-1.443)		1.209 (1.014-1.442)	*<0.001*
Quartiles of AIP					
Q1	1362	1.000 (reference)			
Q2	1288	0.966 (0.594-1.570)	*0.889*	0.847 (0.508-1.414)	*0.526*
Q3	1241	1.068 (0.662-1.723)	*0.787*	0.854 (0.508-1.435)	*0.552*
Q4	1334	1.723 (1.125-2.640	*0.012*	1.461 (0.888-2.403)	*0.136*
*P* for trend			*0.017*		*0.073*

“Model”: adjusted for age, gender, race, education status, family income, current smoking and drinking status, physical activity, BMI, hypertension, diabetes, family history of stroke, and atrial fibrillation. Abbreviations: AIP—atherogenic index of plasma; OR—odds ratio; 95% CI—95% confidence interval.

**Table 3:** Subgroup analyses for the impact of AIP on the risk of ischemic stroke. This multivariate logistic model was adjusted for age, gender, race, education status, family income, current smoking and drinking status, physical activity, BMI, hypertension, diabetes, family history of stroke, and atrial fibrillation.

Age (years)	OR	95% CI	*P* value
<55	1.144	(0.874-1.497)	*0.329*
≥55	1.295	(1.135-1.478)	*<0.001*
HTN			
Yes	1.312	(1.155-1.489)	*<0.001*
No	1.059	(0.761-1.473)	*0.735*
DM			
Yes	1.218	(0.971-1.527)	*0.088*
No	1.302	(1.134-1.495)	*<0.001*
BMI (kg/m^2^)			
<28	1.338	(1.166-1.535)	*<0.001*
≥28	1.098	(0.866-1.392)	*0.440*

Abbreviations: HTN—hypertension; DM—diabetic mellitus; BMI—body mass index; OR—odds ratio.

**Table 4:** The consideration of AIP improved risk stratification for ischemic stroke, as determined by NRI.

Variable	Male	*P* value	Female	*P* value
Conventional risk factors	Reference		Reference	
Conventional risk factors +	0.175 (0.017-0.333)	*<0.297*	0.255 (0.111-0.400)	*<0.001*
AIP				

Abbreviations: AIP—atherogenic index of plasma; NRI—net reclassification improvement. Conventional risk factors included age, education, regular exercise, current smoking, current drinking, atrial fibrillation, hypertension, BMI, family history of stroke, and diabetes.

## Data Availability

Answer: yes. Comment: the datasets used and/or analyzed during the current study do not contain identifiable data and are available from the corresponding author on reasonable request.
